# Spoilage assessment of chicken breast fillets by means of fourier transform infrared spectroscopy and multispectral image analysis

**DOI:** 10.1016/j.crfs.2021.02.007

**Published:** 2021-02-25

**Authors:** Evgenia D. Spyrelli, Onur Ozcan, Fady Mohareb, Efstathios Z. Panagou, George- John E. Nychas

**Affiliations:** a Laboratory of Microbiology and Biotechnology of Foods, Department of Food Science and Human Nutrition, School of Food and Nutritional Sciences, Agricultural University of Athens, Iera odos 75, 11855, Athens, Greece; b Bioinformatics Group, Department of Agrifood, School of Water, Energy and Environment Cranfield University, College Road, Cranfield, Bedfordshire, MK43 0AL, UK

**Keywords:** Chicken breast fillets, Fourier transform infrared spectroscopy, Multispectral imaging, Multivariate data analysis, Machine learning, SorfML platform

## Abstract

The objective of this research was the evaluation of Fourier transforms infrared spectroscopy (FT-IR) and multispectral image analysis (MSI) as efficient spectroscopic methods in tandem with multivariate data analysis and machine learning for the assessment of spoilage on the surface of chicken breast fillets. For this purpose, two independent storage experiments of chicken breast fillets (n ​= ​215) were conducted at 0, 5, 10, and 15 ​°C for up to 480 ​h. During storage, samples were analyzed microbiologically for the enumeration of Total Viable Counts (TVC) and *Pseudomonas* spp. In addition, FT-IR and MSI spectral data were collected at the same time intervals as for microbiological analyses. Multivariate data analysis was performed using two software platforms (a commercial and a publicly available developed platform) comprising several machine learning algorithms for the estimation of the TVC and *Pseudomonas* spp. population of the surface of the samples. The performance of the developed models was evaluated by intra batch and independent batch testing. Partial Least Squares- Regression (PLS-R) models from the commercial software predicted TVC with root mean square error (RMSE) values of 1.359 and 1.029 log CFU/cm^2^ for MSI and FT-IR analysis, respectively. Moreover, RMSE values for *Pseudomonas* spp. model were 1.574 log CFU/cm^2^ for MSI data and 1.078 log CFU/cm^2^ for FT-IR data. From the implementation of the in-house sorfML platform, artificial neural networks (nnet) and least-angle regression (lars) were the most accurate models with the best performance in terms of RMSE values. Nnet models developed on MSI data demonstrated the lowest RMSE values (0.717 log CFU/cm^2^) for intra-batch testing, while lars outperformed nnet on independent batch testing with RMSE of 1.252 log CFU/cm^2^. Furthermore, lars models excelled with the FT-IR data with RMSE of 0.904 and 0.851 log CFU/cm^2^ in intra-batch and independent batch testing, respectively. These findings suggested that FT-IR analysis is more efficient than MSI to predict the microbiological quality on the surface of chicken breast fillets.

## Highlights

• Poultry meat’s vulnerability to spoilage demands rapid quality assessment LWT-Food Sci. Technol.methods.
• FT-IR and MSI are non-invasive methods applied in a variety of meat products.
• SorfML is a web platform providing diverse machine learning algorithms.
• FT-IR analysis via lars predicted efficiently microbial loads of TVC.

## Graphical abstract

Scheme describing data processing and models construction.

**Figure:**
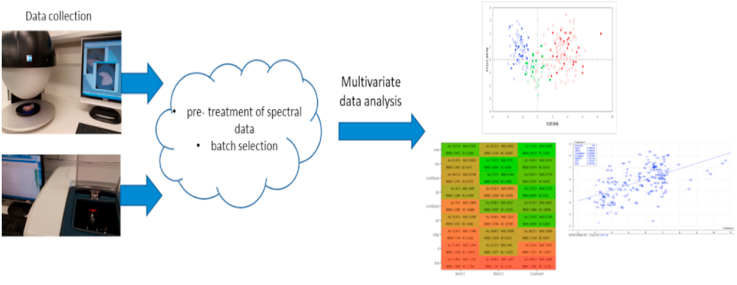

## 1 Introduction

According to the Food and Agriculture Organization (FAO, 2019) around 14 % of the world’s food is lost after harvest and before reaching the retail level, including on-farm activities, storage and transportation. A key to the reduction of food waste is to improve the efficiency of the food system by monitoring each production stage carefully (FAO, 2019). At the same time, consumers’ awareness and demand for high quality and safe food has been continuously arisen, especially in the case of meat products. Poultry meat and more specifically chicken breast is one of the most preferable products due to its high protein content and low price (FAO, 2020). However, its susceptibility to spoilage (Dawson et al., 2013; Rouger et al., 2017; Silva, et al., 2018) necessitates the rapid quality assessment during production, transportation or retail in order to avoid further food waste.

An alternative approach for rapid quality assessment, feasible by technology and science evolution, is the implementation of spectroscopic methods such as vibrational spectroscopy (FT-IR, NIR, Raman) (Argyri et al., 2013; Alamprese et al., 2016; Grassi and Alamprese, 2018), hyperspectral and multispectral imaging (Liu et al., 2014; Qin et al., 2013) and biomimetic sensors (e-nose, e-tongue) (Loutfi et al., 2015; Wojnowski et al., 2017). These nondestructive methods can be combined with microbiological, sensory and multivariate data analysis for the development of models evaluating meat quality. In addition, the developed models accompanied by their datasets could be uploaded and maintained in the cloud, updated constantly with new data in order to be consultative to food industries (Nychas et al., 2016; Tsakanikas et al., 2020).

In the last decade, the performance of instruments based on light emission interaction with the surface according to its chemical and physical properties (Hyper and Multispectral Imaging) or vibrational spectroscopy (FT-IR) has been investigated in the evaluation of quality characteristics of various food commodities (Prieto et al., 2009; Xiong et al., 2015). Both spectroscopic methods have been proved promising and effective for the development of predictive models assessing the quality and microbiological load in many meat products (Pu, et al., 2015). Specifically, for poultry products qualitative models have been constructed and evaluated for the classification of intact chicken breast fillets based on hyperspectral analysis (Yang et al., 2018). Moreover, qualitative as well as quantitative models developed on spectral data (400–1100 ​nm) could determine bacterial counts during spoilage of chicken meat (Feng and Sun, 2013; Feng et al., 2013). Likewise, Alexandrakis et al. (2012) proposed FT-IR as effective method for the discrimination of intact chicken breast muscle during spoilage. The potential of FT-IR to accurately detect spoilage bacteria on the surface of chicken meat has been also confirmed by Ellis et al. (2002).

An important and challenging decision in the development of predictive models with spectral data is the performance of the optimum machine learning algorithm resulting in efficient models that describe more accurately the dynamics of microorganisms during spoilage. Until now, many algorithms have been employed in the rapid assessment of meat quality through several software applications (Chen, et al., 2011; Kamruzzaman et al., 2015; Ropodi et al., 2016). SorfML is a publicly available Web platform that has the flexibility to provide rapid screening of experimental data by allowing the development and validation of a variety of linear and non-linear algorithms (Estelles-Lopez et al., 2017; Manthou et al., 2020). This leverage allows user to investigate data’s tendency, exclude models with poor performance and compare the most accurate ones. Additionally, it enables the comparison of different sensors’ performance in order to facilitate the selection of the most reliable analysis/sensor for food quality assessment.

The aim of this research was (i) to develop models derived from different analytical instruments (FT-IR and MSI) assessing the microbiological quality of chicken breast fillets during storage at isothermal conditions, (ii) to assess the performance of different machine learning algorithms and analytical platforms, based on a commercial software and a publicly available Web platform, to monitor the population dynamics of spoilage microorganisms during storage, and (iii) to infer on the potential and limitations of each analytical tool.

## 2 Materials and methods

### 2.1 Experimental design

Chicken breast fillets (*ca*. 245–280 ​g per fillet) were obtained from a Greek poultry industry and transported under refrigeration immediately to the laboratory. The samples were supplied by the industry in plastic packages (width: 25 ​cm, thickness: 90 ​μm, permeability of ca. 25, 90, 6 ​cm³ m^-2^day^-1^bar^-1^ at 20 ​°C and 50% RH for CO_2_, O_2_ and N_2_, respectively) and stored aerobically at four isothermal conditions (0, 5, 10, 15 ​°C) for up to 480 ​h depending on storage temperature. At regular time intervals, spectral data (FT-IR and MSI) were collected from the surface of chicken meat samples and correlated with microbiological data. Two independent experiments were undertaken with two different chicken meat batches (batch 1: B1; batch 2: B2) and duplicate samples were analyzed from each sampling point and storage temperature. Storage of samples was terminated at 480 ​h ​at 0 ​°C while for the highest storage temperature (15 ​°C) the duration of the experiments was 168 ​h. All samples originated from Ross strains broilers with the same feeding, farming and slaughtering conditions. Feeding was customized by the company and comprised of grain, wheat, maize, soya bean oil and meat and premix for broilers (vitamin and mineral supplement). Chickens were slaughtered after 3 months of age and all stages of production were in compliance to EU regulations (823/2004, 824/2004, 834/2004 and 543/2008).

### 2.2 Microbiological analysis

A slice of 20 ​cm^2^ (maximum thickness: 2 ​mm) from the surface of chicken breast fillet was removed aseptically using a sterile stainless steel cork borer (2.5 ​cm in diameter), scalpel and forceps, added to 100 ​mL of sterile quarter strength Ringer’s solution (Lab M Limited, Lancashire, United Kingdom) and homogenized in a Stomacher device (Lab Blender 400, Seward Medical, United Kingdom) for 120 ​s ​at room temperature. Serial decimal dilutions were prepared in the same medium and 1.0 or 0.1 ​mL of the appropriate dilutions were spread or poured on the following media: a) Tryptic glucose yeast agar (Plate Count Agar, Biolife, Milan, Italy) for the enumeration of Total Viable Counts (TVC) incubated at 25 ​°C for 72 ​h; b) Pseudomonas Agar Base with selective supplement cephalothin-fucidin-cetrimide (LabM Limited, Lancashire, United Kingdom) for the enumeration of *Pseudomonas* spp. after incubation at 25 ​°C for 48 ​h. After incubation, typical colonies for each microbial group were enumerated and colony counts were logarithmically transformed and expressed as log CFU/cm^2^. Further on, the primary model of Baranyi and Roberts (1994) was fitted to the growth data of TVC and *Pseudomonas* spp. to determine the kinetic parameters of microbial growth (maximum specific growth rate: μ_max_; lag phase duration).

### 2.3 Gas composition

Prior to microbiological analysis, the gas composition in the headspace of the packages was analyzed using a Dansensor CheckMate 9900 gas analyzer (PBI-Dansensor A/S, Ringsted, Denmark) to monitor the changes in the concentration (%) of O_2_ and CO_2_ during storage.

### 2.4 Spectra acquisition

#### 2.4.1 Multispectral image analysis

MSI spectra were captivated via Videometer-Lab instrument (Videometer A/S, Herlev, Denmark) which frames surface reflectance of samples from 18 different monochromatic wavelengths (405–970 ​nm), namely: 405, 435, 450, 470, 505, 525, 570, 590, 630, 645, 660, 700, 850, 870, 890, 910, 940 and 970 ​nm. The organology of this sensor and the image acquisition is thoroughly described in previous publications (Dissing et al., 2013; Fengou et al., 2019). The result of the measurement is a data cube comprised of spatial and spectral data for each sample of size m×n×18 (where m×n is the image size in pixels) (Tsakanikas et al., 2015). Furthermore, a segmentation process is required for the selection of the Region of interest (ROI) on the samples surface. This process is accomplished by Canonical Discriminant Analysis (CDA) and it is implemented by Videometer-Lab version 2.12.39 software (Videometer A/S, Herlev, Denmark).

#### 2.4.2 FT-IR spectroscopy

FT-IR measurements were performed using a ZnSe 45 HATR (Horizontal Attenuated Total Reflectance) crystal (PIKE Technologies, Madison, Wisconsin, United States), and a FT-IR-6200 JASCO spectrometer (Jasco Corp., Tokyo, Japan). The measurement crystal shows a refractive index of 2.4 and a depth of penetration of 2.0 ​μm ​at 1000 ​cm^-1^. Spectra were obtained at the wavenumber range of 4000 to 400 ​cm^-1^ using Spectra Manager Code of Federal Regulations (CFR) software version 2 (Jasco Corp., Tokyo, Japan), by accumulating 100 scans with a resolution of 4 ​cm^-1^ and a total integration time of 2 ​min.

### 2.5 Data analysis

#### 2.5.1 PLS-R unscrambler

For the development of PLS-R models assessing TVC and *Pseudomonas* spp. counts the statistical software Unscrambler © ver.9.7 (CAMO Software AS, Oslo, Norway) was used. Prior to analysis, MSI data were pre-treated by Standard Normal Variate (SNV) transformation for the exclusion of collinear and “noisy” data (Bi et al., 2016). Likewise, FT-IR spectral data were subjected to Savinsky-Golay pre-treatment (second polynomial order, 1^st^ derivative, 9-point window) (independent variables ​= ​829) to minimize baseline shifts and noise (Rinnan et al., 2009; Alamprese et al., 2016). Additionally, wavenumbers in the range of 900–2000 ​cm^-1^ were utilized for the analysis as suggested by other researchers (Argyri et al., 2013; Ropodi et al., 2018). Calibration and full cross validation (leave-one-out cross validation) were conducted using one batch (n ​= ​115) and prediction was implemented by the second batch (n ​= ​99). Independent variables for PLS-R models were the spectral data acquired by MSI and FT-IR and TVC and *Pseudomonas* spp. counts were considered as dependent variables.

#### 2.5.2 Using SorfML for model development and validation

An alternative approach was investigated by the implementation of the sorfML software (www.sorfml.com), in which nine algorithms were considered for the prediction of TVC counts, namely Partial-least squares (pls) (Geladi and Kowalski, 1986); Support vector machine with linear kernel (svmLinear) (Cortes and Vapnik, 1995); Support vector machine with radial basis function kernel (svmRadial); Random forests (rf) (Breiman, 2001); K-nearest neighbours (knn) (Cover and Hart, 1967); Principal component regression (pcr) (Jolliffe, 1982); Least-angle regression (lars) (Loubes and Massart, 2004); Ridge regression (ridge) (Hoerl and Kennard, 1970); Artificial neural network (nnet) (Jain et al., 1996). Spectral data were mean-centered and standardized prior to analysis. This modification allows every variable equal opportunity to influence the final statistical model (Verboven et al., 2012). FT-IR spectral data set was constricted from 800 to 4000 ​cm^-1^.

**Fig. 1:**
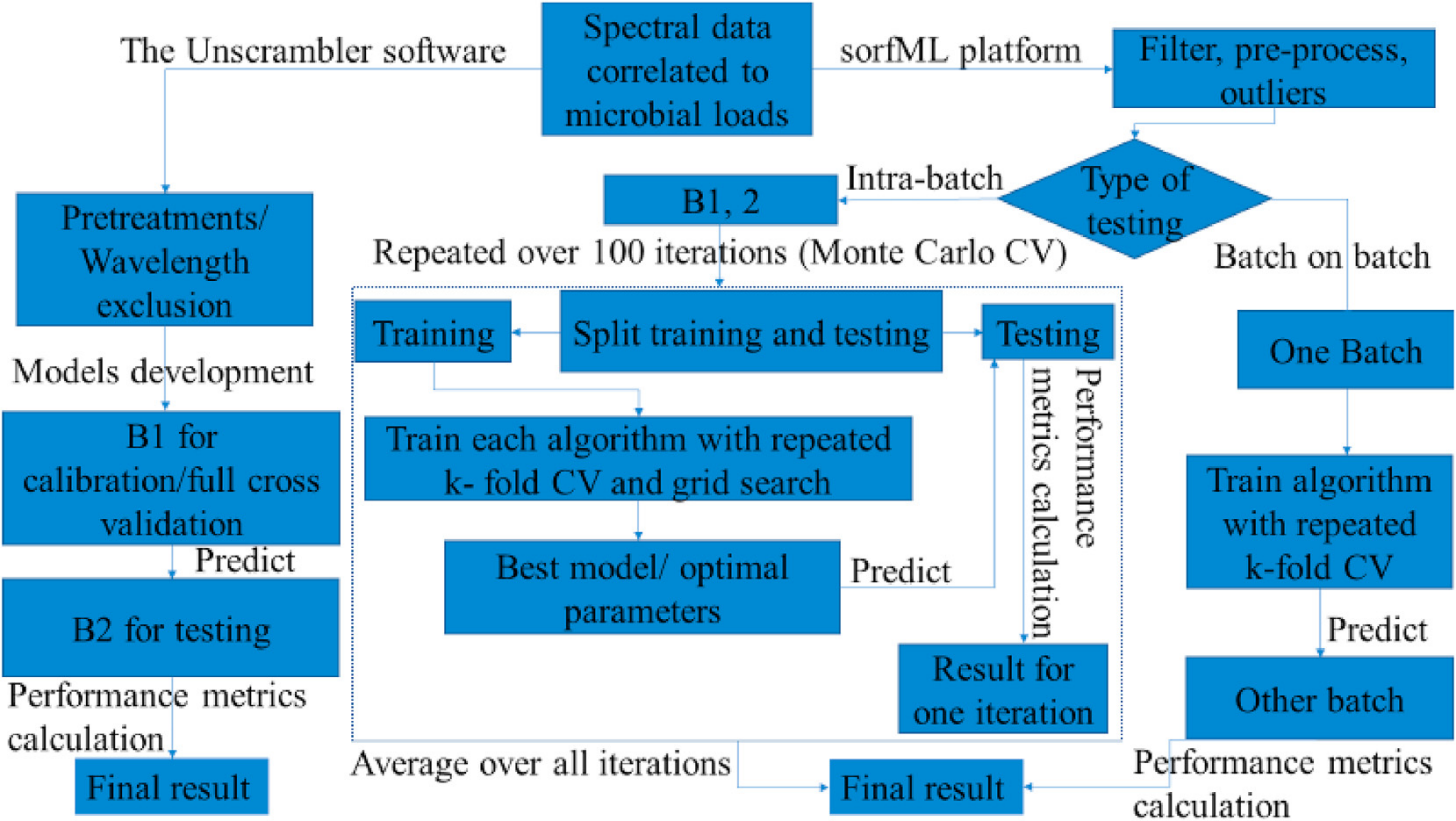
Flowchart describing model’s development and validation though The Unscrambler and sorfML via data processing stage.

Another point of attention in the sorfML software analysis was the splitting procedure of the data sets, which consisted of two phases (Fig. 1). In the first one, the dataset (one batch) was separated randomly into training and testing sets with a 70%–30% split. Each machine learning algorithm was applied to the training set using repeated k-fold cross validation (k ​= ​10, repeats ​= ​3) and grid search to obtain best performing models with the optimal parameters. After model development, prediction was undertaken by the test set to assess overall performance which is firmly depended on the random training/test split undertaken. In order to provide an appropriate and unbiased outcome, Monte Carlo cross validation was implemented (k ​= ​100) for a number of times with different training and test splits, and giving an average of the performance of all iterations (Xu and Liang, 2001). In the second phase, one batch was trained with k-fold cross validation (k ​= ​10, repeats ​= ​3) and the best model was validated on the other batch (B1 on B2: B1 as training set and B2 as testing one; B2 on B1: B2 as training set and B1 as testing one).

### 2.6 Model performance indexes

The assessment of model performance was based on the calculation of the root mean squared error (RMSE) (Sant’Ana, Franco & Schaffner, 2012; Feng et al., 2013), mean absolute error (MAE) (Sang, Lü, Zeng, Zhang & Zhou, 2008), coefficient of determination (R^2^) (Asuero et al., 2006) and accuracy index. Unlike classification models, accuracy in the case of quantitative models could be defined as TVC predictions within 1 log CFU/cm^2^ off the actual (observed) values (Estelles-Lopez et al., 2017). Supplementary to these metrics, r (correlation coefficient) was computationally calculated via the Unscrambler software. Eventhough the above mentioned performance metrics were calculated, models accuracy on prediction was assessed based on RMSE values.

## 3 Results

### 3.1 Microbiological analysis

**Fig. 2:**
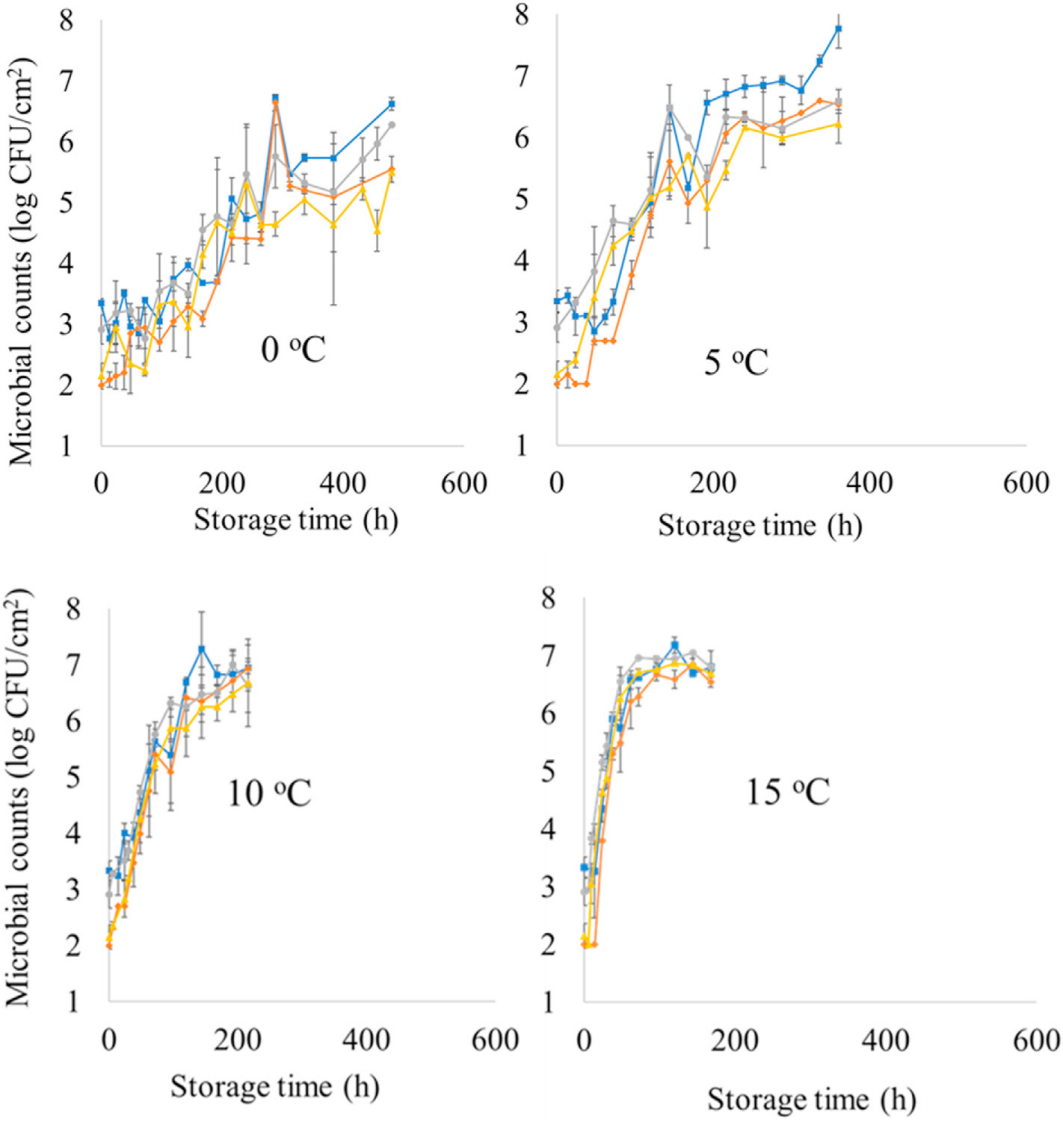
Microbial counts of TVC (batch 1: blue line), *Pseudomonas* spp. (batch 1: orange line), TVC (batch 2: grey line) and) and *Pseudomonas* spp. (batch 2: yellow line) on the surface of chicken breast fillet samples stored at 0, 5, 10 and 15 ​°C. (For interpretation of the references to color in this figure legend, the reader is referred to the Web version of this article.)

The microbial population of TVC and *Pseudomonas* spp. on the surface of chicken breast fillets for each storage condition is presented in Fig. 2. The initial load of TVC was 3.3 and 2.9 log CFU/cm^2^ in B1 and B2, respectively. Likewise, *Pseudomonas* spp. was enumerated at the beginning of storage at 2.0 and 2.1 log CFU/cm^2^ for B1 and B2, respectively. Storage temperature seemed to significantly influence the growth of chicken’s microbiota as inferred by the respective kinetic parameters for TVC and *Pseudomonas* spp. as derived by the primary growth model of Baranyi and Roberts (1994) (supplementary material, Table A). Specifically, the lag phase duration and μ_max_ of *Pseudomonas* spp. of chicken samples stored at 0 ​°C were 72.2 ​h and 0.036 h^-1^, respectively. On the contrary, samples stored at 15 ​°C exhibited μ_max_ and lag phase duration of *Pseudomonas* spp. at 0.241 h^-1^ and 8.8 ​h, respectively.

TVC and *Pseudomonas* spp. counts in B1 and B2 presented variations during storage at 0 and 5 ​°C but always within the range of ± 1 log unit. At the end of storage, TVC and *Pseudomonas* spp. counts on samples from B1 were 6.2 and 5.7 log CFU/cm^2^, respectively. Similarly, for B2 samples the level of final TVC and *Pseudomonas* spp. counts was 6.3 and 5.5 log CFU/cm^2^, respectively. For B1 at 5 ​°C the number of TVC and *Pseudomonas* spp. after a period of 360 ​h was 6.8 and 6.6 log CFU/cm^2^, respectively, while for B2 at the same storage conditions, TVC and *Pseudomonas* spp. counts were 7.6 and 6.2 log CFU/cm^2^, respectively. This difference in microbial counts was expected as samples of B1 and B2 were collected with an interval of 4 months (winter-spring) to take into account seasonal variation. It is also worth noting that in all storage conditions, the final number of TVC ranged between 6.2-7.6 log CFU/cm^2^, unlike other studies reporting spoilage level of poultry meat at 7.0–8.0 log CFU/cm^2^ (Rouger et al., 2017). The lower TVC counts during spoilage of poultry meat observed in this work could be attributed to the non-permeable film used by the poultry company as packaging material. Indeed, the percentage of CO_2_ inside the packages at the end of storage was 14.3 % and 47.5 % for samples stored at 0 ​°C and 15 ​°C, respectively (Supplementary material, Fig. A).

### 3.2 Spectral measurements

**Fig. 3:**
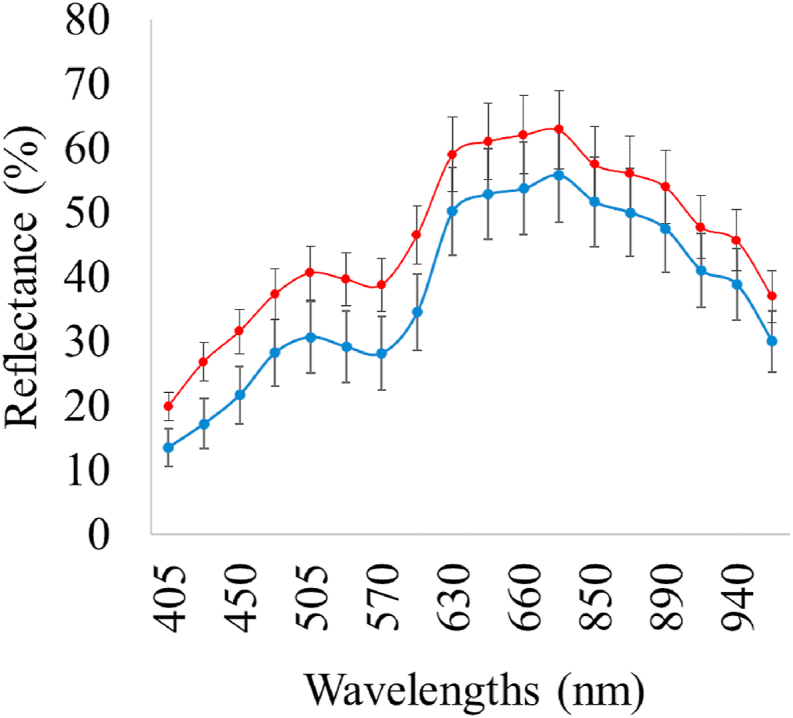
Spectrum of fresh (blue line, storage time: 0 ​h) and spoiled (red line, storage time: 456 ​h) chicken breast fillet samples stored at 0 ​°C from MSI spectra (wavelengths: 405–970 ​nm). (For interpretation of the references to color in this figure legend, the reader is referred to the Web version of this article.)

**Fig. 4:**
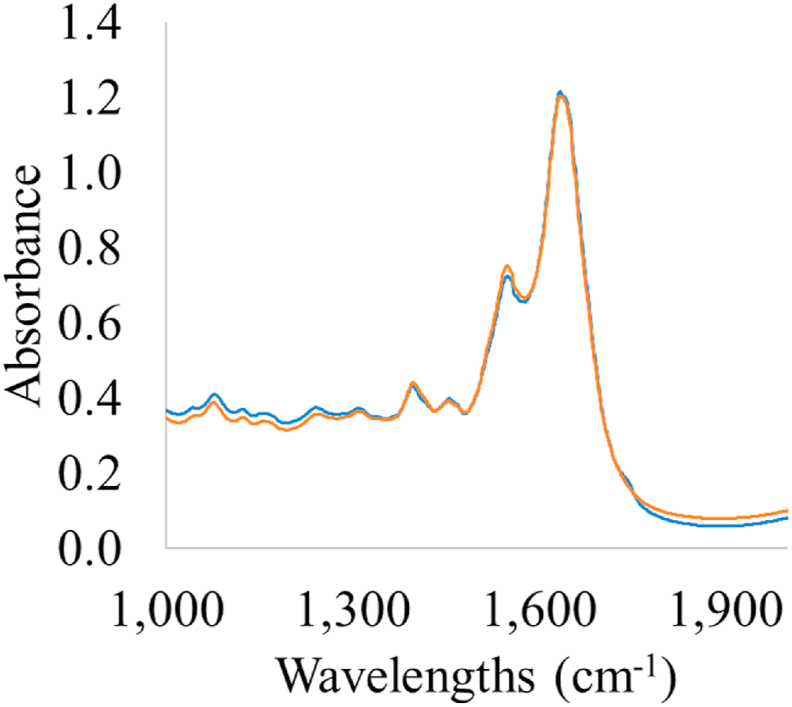
Spectrum of fresh (blue line, storage time: 0 ​h) and spoiled (red line, storage time: 456 ​h) chicken breast fillet samples stored at 0 ​°C from FT-IR measurements (wavelengths: 1000- 2000 ​cm^-1^). (For interpretation of the references to color in this figure legend, the reader is referred to the Web version of this article.)

Typical MSI and FT-IR spectra of fresh (0 ​h corresponding to 3.3 log CFU/cm^2^) and spoiled (456 ​h corresponding to 5.9 log CFU/cm^2^) chicken breast fillet samples are illustrated in Fig. 3, Fig. 4, respectively. The comparison of reflectance in MSI spectra between fresh and spoiled samples confirmed the role of myoglobin in meat color assessment (570–700 ​nm). Concerning FT-IR spectra, the contribution of the absorption bands in the range of 1400–1800 ​cm^-1^ for the prediction of the microbial counts on the surface of samples is highlighted in Fig. 4. The absorbance in this region is mainly related to the metabolic fingerprint of samples which is derived from the metabolic activity of microorganisms during spoilage procedure (Alexandrakis et al., 2012).

### 3.3 Models assessing microbial population via MSI analysis

**Fig. 5:**
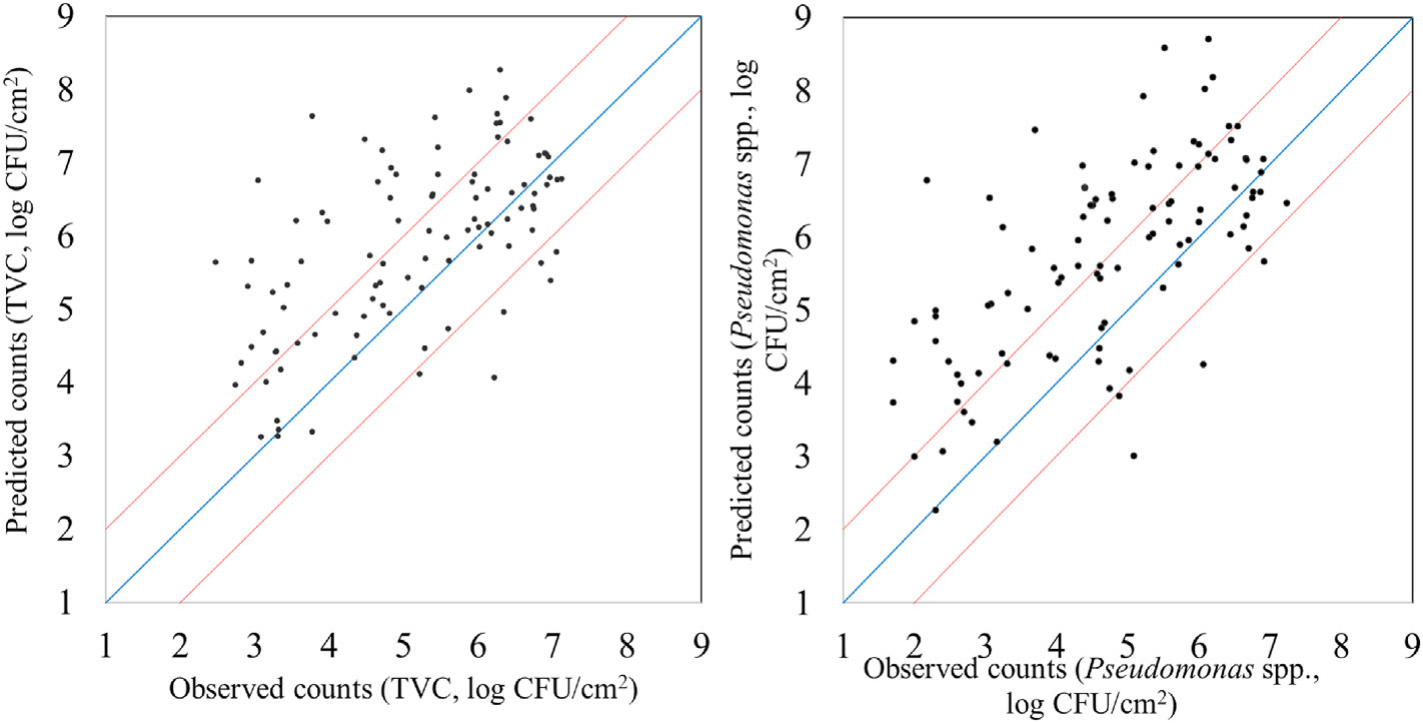
Predicted versus observed TVC and *Pseudomonas* spp. counts after MSI models validation. Blue line depictures the line of equity (y ​= ​x) and red lines indicate ​± ​1 log unit area. (For interpretation of the references to color in this figure legend, the reader is referred to the Web version of this article.)

**Fig. 6:**
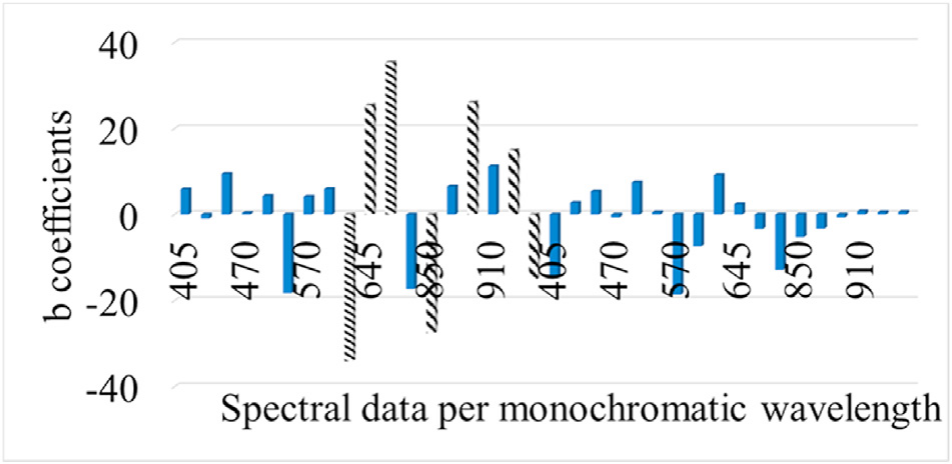
b coefficients of PLS-R model for MSI analysis per monochromatic wavelength from 405 to 970 ​nm. Dashed bars represent data per wavelength that influenced more model’s performance.

Performance metrics (r, RMSE, R^2^, MAE, accuracy) as well as linear parameters (slope, offset) are provided in Table 1 for PLS-R model calibration, cross-validation and prediction, estimating the level of TVC and *Pseudomonas* spp. on the surface of chicken breast fillets via MSI analysis. More specifically, RMSE and r values ranged between 0.752-1.359 log CFU/cm^2^ and 0.604–0.876, respectively for the estimation of TVC counts when B1 was used as training set and B2 as testing set. Similar performance was observed for PLS-R model assessing *Pseudomonas* spp. counts. In this case, the values of r increased from 0.665-0.905, while RMSE exhibited values in the range of 0.724–1.574 log CFU/cm^2^. Additionally, a graphical approach of these linear models is represented in Fig. 5 where predicted vs observed TVC and *Pseudomonas* spp. counts are illustrated. Beta coefficients of the models are provided in order to comprehend the contribution of specific wavelengths to model development. As demonstrated in Fig. 6, six of the 36 spectral variables were important in model optimization as their beta coefficients significantly differed from those of the other wavelengths. Wavelengths influencing PLS-R model were 630, 645 and 660 ​nm. Likewise, high values of b coefficients noticed at 850, 890 and 940 ​nm.

**Table 1:** MSI model performance parameters (slope, offset, Latent variables LVs,) and metrics (r, RMSE, R^2^, MAE, Accuracy %).

TVC	N	LVs	slope	offset	Correlation coefficient r	RMSE	R^2^	MAE	% Accuracy
Calibration	115^a^	9	0.768	1.177	0.876	0.752	0.768		
FCV	115^a^	9	0.719	1.428	0.807	0.931	0.651		
Prediction	100^b^		0.534	3.139	0.604	1.359	-0.025	1.042	59
*Pseudomonas* spp.									
Calibration	115^a^	10	0.818	0.817	0.904	0.724	0.818		
FCV	115^a^	10	0.766	1.035	0.843	0.920	0.712		
Prediction	100^b^		0.597	2.930	0.664	1.574	-0.117	1.276	51

a Data set from batch 1.

b Data set from batch 2; LVs: Latent variables; FCV: Full-cross validation.

**Fig. 7:**
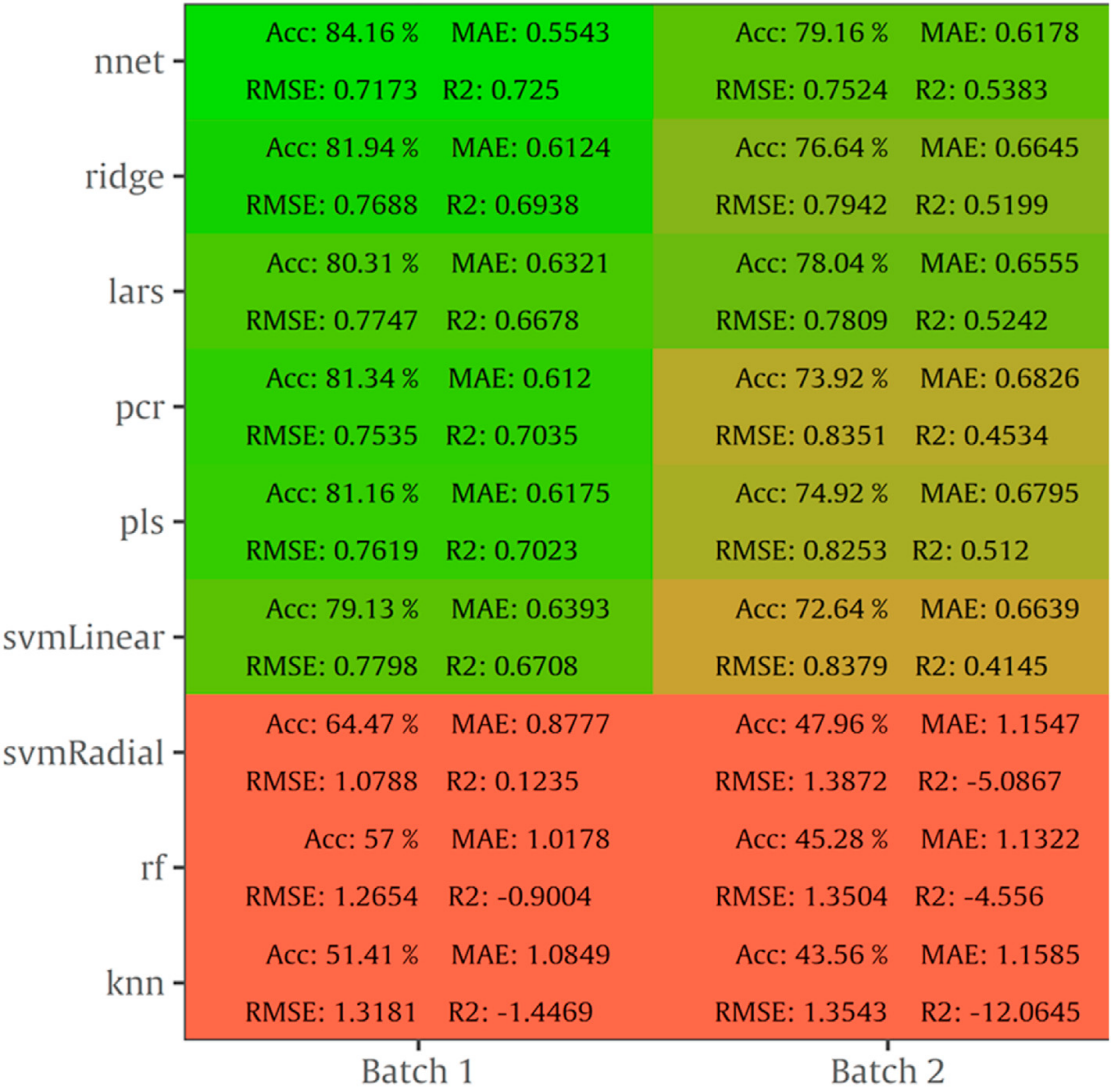
Performance metrics (Accuracy, MAE, RMSE, R^2^) of MSI models with intra-batch validation.

Results for MSI spectral data after the implementation of 9 algorithms via sorfML platform consisting of internal testing on B1 and B2, averaged over 100 iterations (Monte Carlo cross validation) are shown in Fig. 7. RMSE values ranged from 0.717 to 1.387 log CFU/cm^2^, MAE from 0.554 to 1.158, R^2^ from -12.064 to 0.725 and accuracy from 43.5 % to 84.1 %. The highest performance was achieved with nnet with RMSE value of 0.717 log CFU/cm^2^ on B1 and 0.752 log CFU/cm^2^ on B2. Additionally, other machine learning algorithms such as ridge, lars, pcr, pls and svmLinear performed equally well with RMSE values below 0.78 log CFU/cm^2^.

**Fig. 8:**
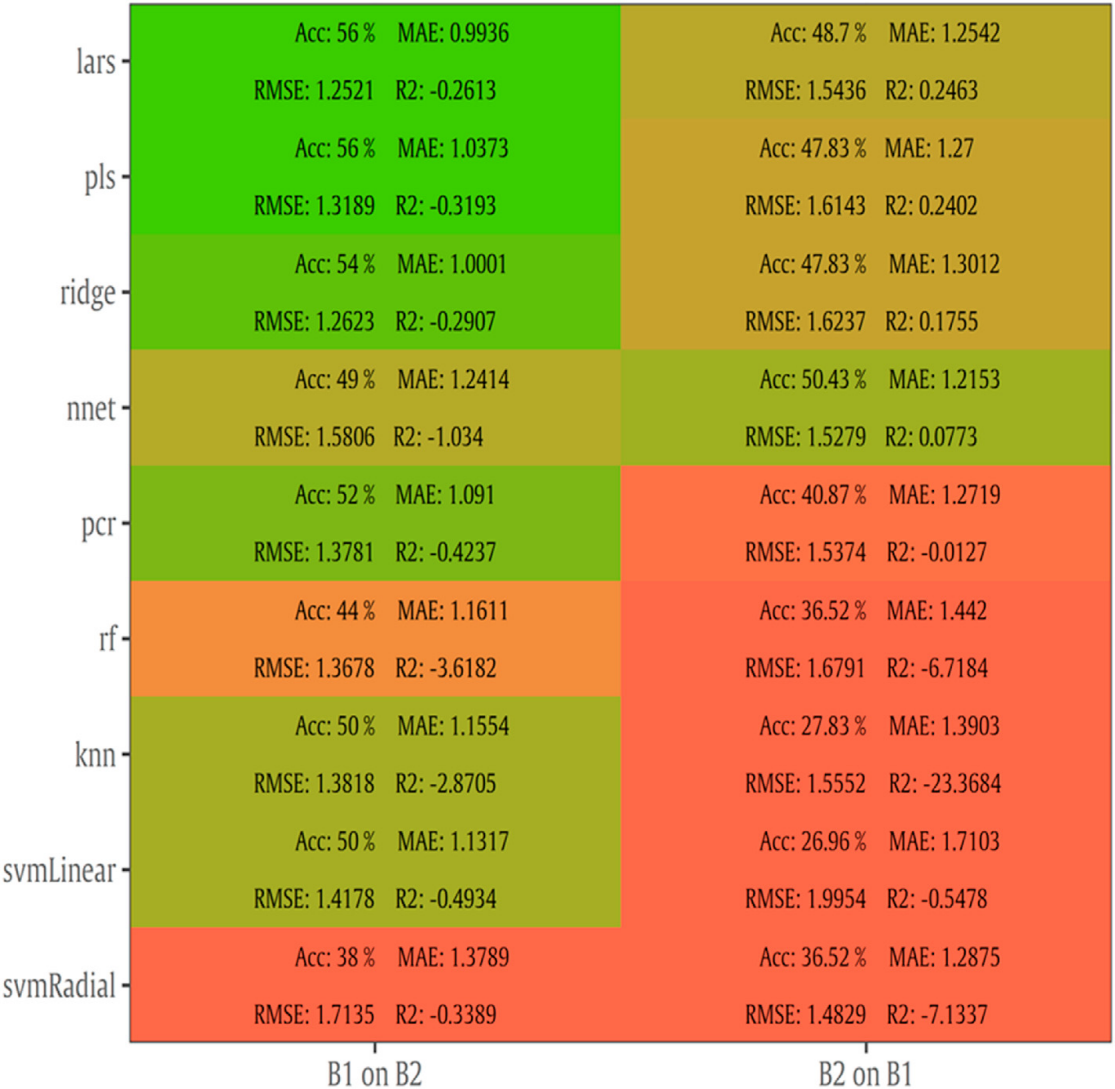
Performance metrics (Accuracy, MAE, RMSE, R^2^) of MSI models with batch-on-batch validation. Model B1 on B2 was developed via batch 1 and tested via batch 2. The reversed procedure was followed for B2 on B1.

Following the same approach, results for batch-on-batch are provided in Fig. 8. A less satisfactory performance can be observed compared to intra-batch testing, with RMSE values ranging from 1.252 to 1.995 log CFU/cm^2^, MAE from 0.993 to 1.710, R^2^ from -23.368 to 0.246 and accuracy from 27 to 56 %. More specifically, the models developed on B1, predicted TVC population from B2 with around 0.3 higher performance on RMSE values. In contrast to intra-batch case, the highest performance was accomplished by lars with RMSE of 1.252 log CFU/cm^2^. Model’s optimization with B1 exhibited low values of RMSE (1.251 versus 1.544 log CFU/cm^2^ for lars model). However, in the case of B2 as a calibration data set, R^2^ values presented improved values, especially when lars, pls and ridge algorithms were applied.

### 3.4 Models assessing microbial population via FT- IR analysis

**Fig. 9:**
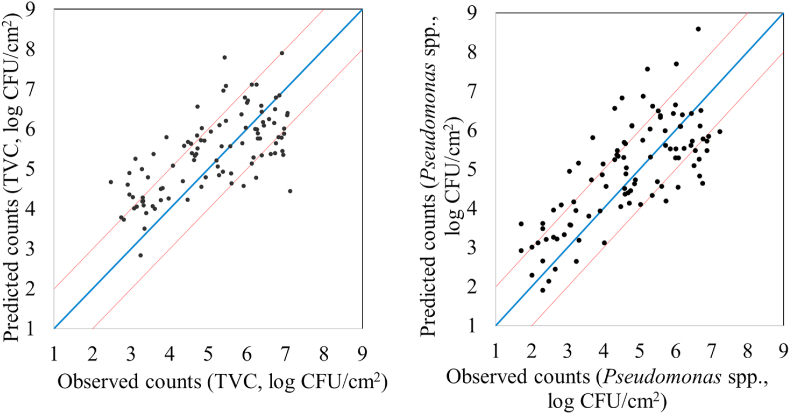
Predicted versus observed TVC and *Pseudomonas* spp. counts after FT-IR models validation. Blue line depictures the line of equity (y ​= ​x) and red lines indicate ​± ​1 log unit area. (For interpretation of the references to color in this figure legend, the reader is referred to the Web version of this article.)

**Fig. 10:**
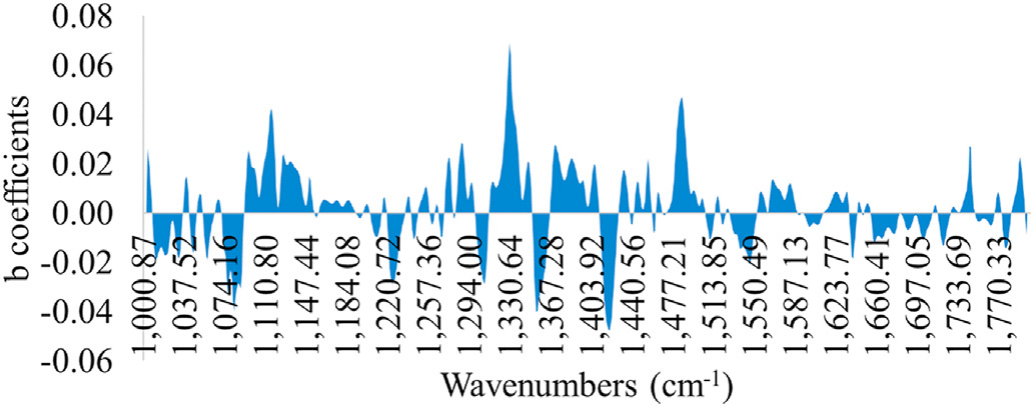
b coefficients of PLS-R model for FT-IR analysis for each wavelength within 1000–1800 ​cm^-1^.

**Fig. 11:**
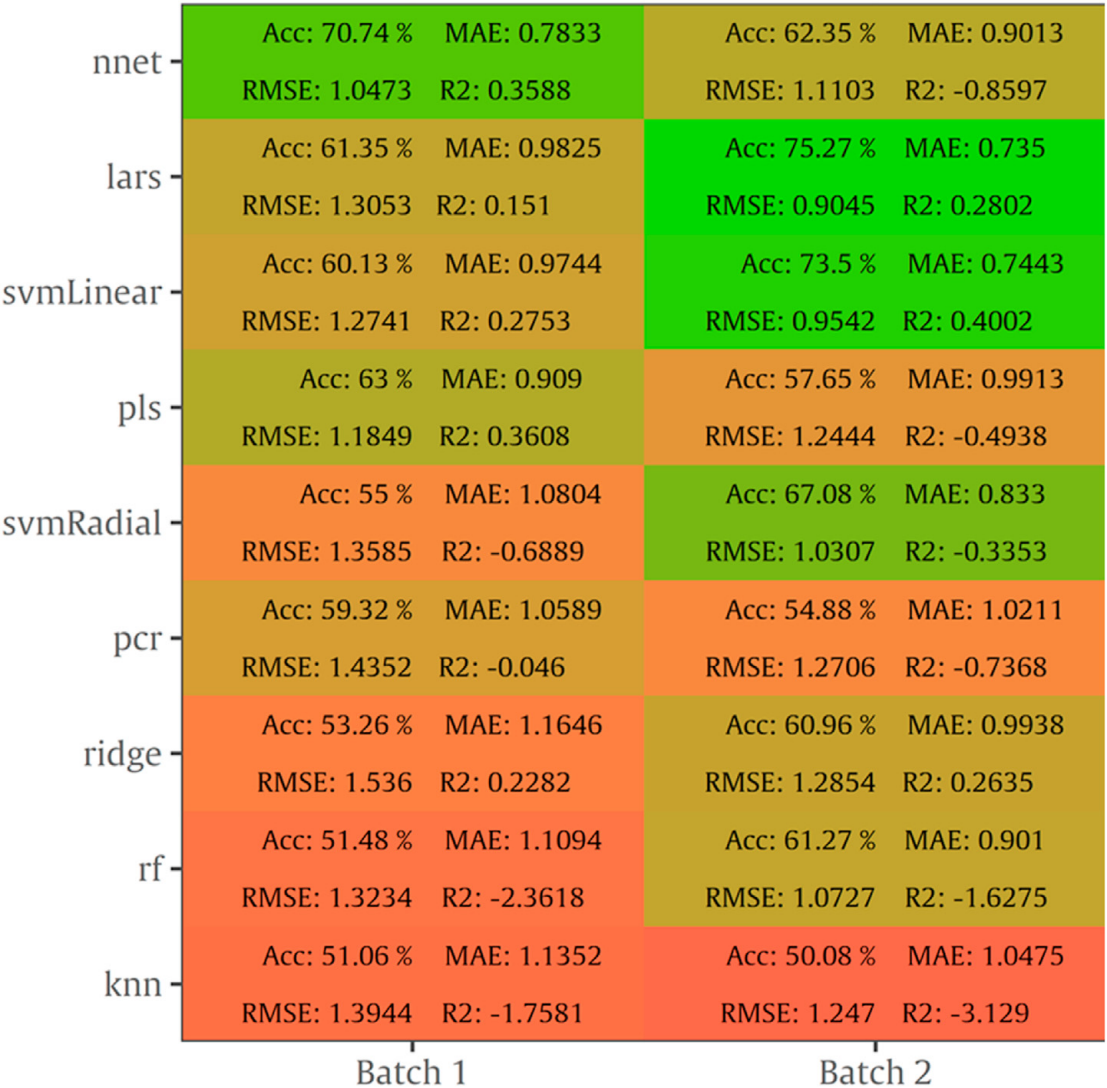
Performance metrics (Accuracy, MAE, RMSE, R^2^) of FT-IR models with intra-batch validation.

**Fig. 12:**
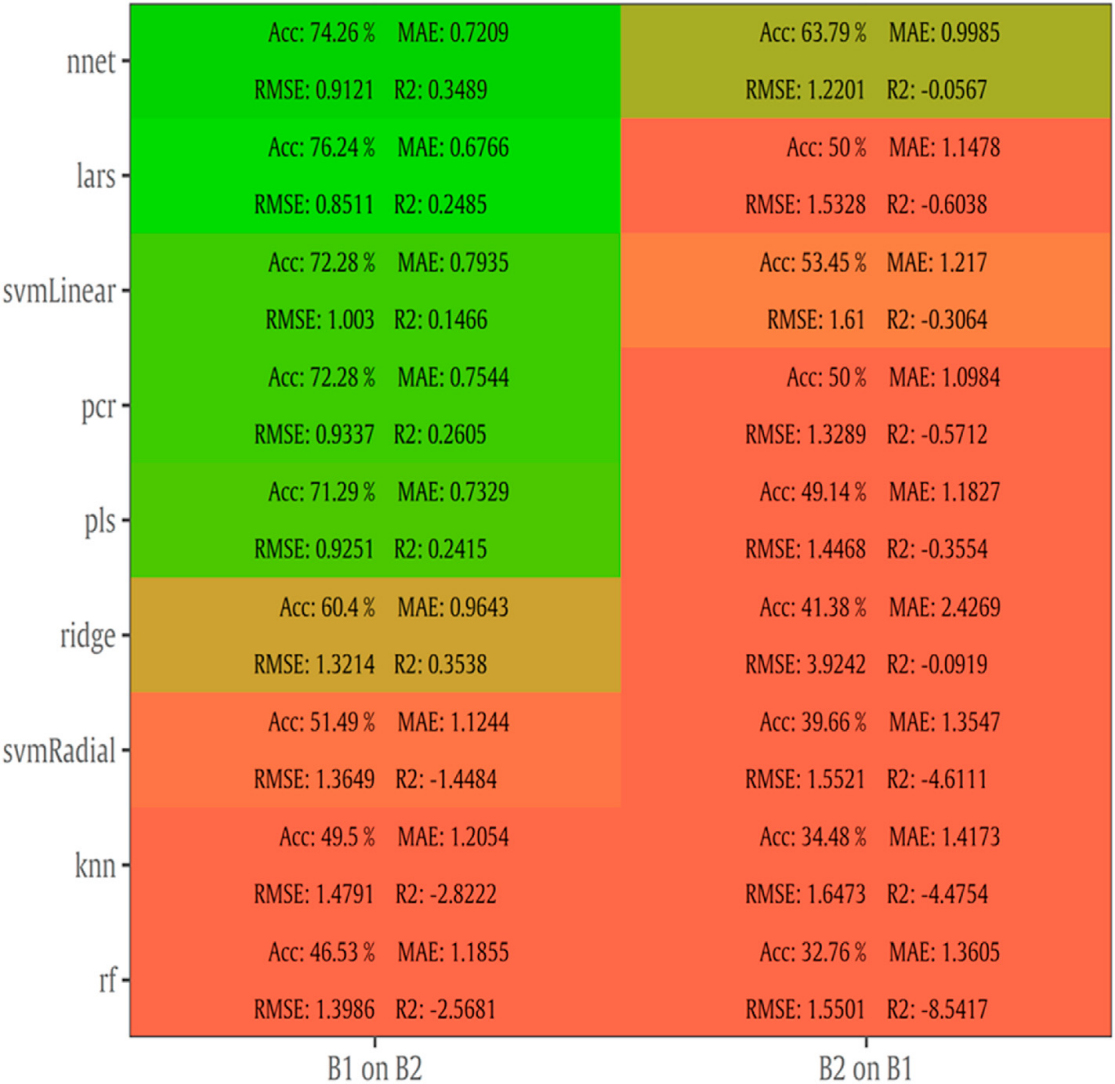
Performance metrics (Accuracy, MAE, RMSE, R^2^) of FT-IR models with batch-on-batch validation. Model B1 on B2 was developed via batch 1 and tested via batch 2. The reversed procedure was followed for B2 on B1.

The findings of models predicting TVC and *Pseudomonas* spp. counts with FT-IR measurements are shown in Fig. 9, Fig. 10, Fig. 11, Fig. 12. Performance metrics for PLS-R models are also provided in Table 2 for calibration, cross-validation and prediction procedures where B1 was used for model development and B2 for testing. For the estimation of TVC on chicken breast, RMSE and r demonstrated values 0.739–1.029 log CFU/cm^2^ and 0.679–0.882, respectively. PLS-R model for *Pseudomonas* spp. via FT-IR exhibited r values of 0.739–0.916 and RMSE values were from 0.683 to 1.077 log CFU/cm^2^. The influence of each spectral variable is illustrated in Fig. 10 in terms of beta coefficients of the PLS-R models per wavenumber. The main region between 1004 to 1222 ​cm^-1^ contained interesting information and therefore had great impact on model development. Absorption bands of 1230–1403 ​cm^-1^ were considered as important for the prediction of TVC and *Pseudomonas* spp. Beta coefficients of 1432–1498 ​cm^-1^ as well as 1549- 1584 ​cm^-1^ and 1658- 1704 ​cm^-1^ had impact on model construction.

**Table 2:** FT-IR model performance parameters (slope, offset, Latent variables LVs,) and metrics (r, RMSE, R^2^, MAE, Accuracy %).

TVC	N	LVs	slope	offset	Correlation coefficient r	RMSE	R^2^	MAE	% Accuracy
Calibration	115^a^	5	0.777	1.128	0.882	0.739	0.777		
FCV	115^a^	5	0.654	1.805	0.778	0.989	0.609		
Prediction	99^b^		0.493	2.883	0.679	1.029	0.416	0.861	65
*Pseudomonas* spp.									
Calibration	115^a^	5	0.839	0.723	0.916	0.683	0.839		
FCV	115^a^	5	0.669	1.528	0.749	1.155	0.549		
Prediction	99^b^		0.682	1.767	0.739	1.077	0.481	0.894	65

a Data set from batch 1.

b Data set from batch 2; LVs: Latent variables; FCV: Full-cross validation.

The results for intra batch training for FT-IR data are summarized as a heatmap in Fig. 11 containing also the performance metrics for the 9 algorithms. RMSE values ranged from 0.857 to 1.536 log CFU/cm^2^, MAE from 0.669 to 1.164, R^2^ from -3.129 to 0.546, and accuracy from 50.0 to 75.9 %. As Fig. 11 indicates, prediction on B2 was more accurate than B1 based on RMSE values. Nnet exhibited acceptable performance on B1 with 1.047 log CFU/cm^2^ for RMSE, while lars and svmLinear algorithms performed better with RMSE being at 0.904 and 0.954 log CFU/cm^2^, respectively for B2.

Likewise, batch-on-batch prediction metrics are represented in Fig. 12. In comparison to MSI models, FT-IR models predicted TVC counts satisfactory when B1 was used as training set. RMSE values ranged from 0.851 to 3.924 log CFU/cm^2^ while training model on B1 and validating on B2 outperformed the second model around significantly with 55% lower RMSE. More specifically, nnet accomplished the lowest RMSE (0.851 log CFU/cm^2^) and MAE (0.67 log CFU/cm^2^) over the other algorithms as well as models trained on B2 and validated on B1.

## 4 Discussion

The initial population of TVC and *Pseudomonas* spp. was 3.1 (±0.29) and 2.1 (±0.15) log CFU/cm^2^, respectively, which is considered low compared to published data where the respective counts for TVC and *Pseudomonas* spp. were above 5.0 and 3.5 log CFU/cm^2^, respectively (EFSA, 2016; Rouger et al., 2017). As presented in Fig. 2, the final population of microbiota was considerably low in the case of samples stored at 0 ​°C in comparison to the threshold of spoilage of other meats (*ca.* 7.0–8.0 log CFU/cm^2^) (Nychas et al., 2008). Unlike literature (Rouger et al., 2017), *Pseudomonas* spp. counts were enumerated at the final sampling point at 0 ​°C below 7 log CFU/cm^2^ (Al-Nehlawiet al., 2013), due to the fact that packaging film did not permit diffusion of gases. Therefore, the produced CO_2_ from microbiota’s metabolic reactions acted as modified atmosphere packaging (Koutsoumanis et al., 2008; Liang et al., 2012; Holl et al., 2016). The differences between and within batches could be attributed to animals’ variations (Marcato et al., 2006), alterations of nutrition (Sakomura et al., 2015) as well as by the time of the year (winter-summer), slaughtering and distributing to retail points (Nychas et al., 2008; Collins et al., 2015). It is worth noting that the 2 analyzed chicken breast fillet samples per sampling point could not be from the same chicken as they were randomly selected.

For MSI spectral data, model performance metrics predicted RMSE from 0.739 to 1.536 log CFU/cm^2^. For the prediction of TVC and *Pseudomonas* spp. counts with PLS-R models, RMSE was 1.359 and 1.574 log CFU/cm^2^, respectively. It needs to be noted that all developed models presented the tendency of overestimating the predicted counts. The increased RMSE values could be further improved (reduced) by applying alternative algorithms and sample splitting. Indeed, the assessment of TVC counts by sorfML platform showed satisfactory results, especially in the case of intra-batch validation and nnet algorithm. In this model, RMSE presented the lowest value (0.717 log CFU/cm^2^) while for ridge model RMSE was 0.769 log CFU/cm^2^. On the contrary, for batch-on-batch validation, three algorithms were considered acceptable for the evaluation of TVC counts, with lars model having RMSE of 1.252 log CFU/cm^2^ followed by pls and ringle models with1.319 and 1.262 log CFU/cm^2^, respectively.

FT-IR models showed satisfactory prediction of counts, with performance metrics achieving better values than MSI. For PLS-R models, TVC and *Pseudomonas* spp. counts were predicted with RMSE being 1.029 and 1.078 log CFU/cm^2^, respectively. For intra batch testing, nnet algorithm for B1 and lars for B2 were considered effective for the evaluation of TVC counts, with lars having lower RMSE (0.905 log CFU/cm^2^) than nnet (1.047 log CFU/cm^2^). In contrast, in batch-on-batch validation, RMSE value for nnet (B1 on B2: 0.912 log CFU/cm^2^) were higher than lars where RMSE had the lowest value (0.851 log CFU/cm^2^).

The differentiation of model performance for the 2-sensor analysis highlights the important role of splitting process, data set selection and algorithm during model’s optimization. One significant factor for accurate prediction is inter-batch variability. Moreover, MSI results on intra-batch performance and its low RMSE suggested that this analysis could be applicable for internal validation or quality control in the production line. The latter option has been confirmed via experiments performed in the production line of chicken products at industrial level (Spyrelli et al., 2020). Furthermore, the fundamental role of training and testing data set definition is demonstrated by FT-IR lars model during B1 on B2 validation, which significantly outperformed batch-on-batch performance of MSI (RMSE: 0.851 vs 1.251 log CFU/cm^2^). Additionally, several models of FT-IR were able to attain respectable prediction on different data sets.

Another step affecting model’s performance is the selection of the appropriate cross-validation procedure. Leave-one out cross validation (LOOCV) implemented for PLS-R models is a variant of *k-*fold cross-validation which removes only one sample at a time from the training set and considers it as a test set. Subsequently, for this case *k* is equal to the number of objects. This method may be useful for small database size presenting the problem of the inability to divide the data set into fairly sized subsets for training and test sets. However, this cross-validation approach can lead to overfit when the sample size is not large enough, and thus, results in high prediction error (Beruetta et al., 2007). In contrast, *k*-fold validation separates training data into *k* random groups, trains the model on *k*-1 groups and evaluates it on the remaining group. This is iterated for each unique group, and for repeated k-fold cross validation, the whole process is repeated for the specified times. Overlapping within training and testing data set was avoided (k ​= ​100) with Monte Carlo cross validation by repeating the process outlined above for a number of times with different training and test splits and by averaging the performance of all iterations (Xu and Liang, 2001). Regarding machine learning algorithms implemented for intra- and batch on batch models, artificial neural network (nnet) and least-angle regression (lars) exhibited better performance metrics overall than other models. The former algorithm is considered as a suitable for spectral data sets due to its high tolerance to noisy data. On the other hand, the accuracy of lars might be explained by its ability in dealing with correlated predictors which are abundant in the existing datasets. Moreover, overfitting could be eliminated by reducing predictors range while simultaneously this reduction could lead to an increasement of the generalising ability of the models (Hesterberg et al., 2008).

The influence of certain wavelengths to MSI model development was documented via b coefficient values for PLS-R models (Fig. 6). Reflectance intensity at 570–700 ​nm is related to the presence of respiratory pigments such as myoglobin (570 ​nm), oxymyoglobin (590 ​nm) and metmyoglobin (630 ​nm) (Panagou et al., 2014; Pu et al., 2015). Fatty acids and fat within the food matrix were mainly responsible for the intensity at 928 and 940 ​nm while reflectance at 910 ​nm is evidence of protein denaturation (Kamruzzaman et al., 2015; Ropodi et al., 2018). Proteins and proteolysis products are in abundance in chicken meat, especially in chicken breast (Lin et al., 2011) and hence absorption band at 910 ​nm is considered as one of the most significant wavelengths for quality assessment on chicken breast fillets. Moreover, O–H second overtones observed at 750 and 970 ​nm are related to the moisture content in the raw samples (Dixit et al., 2017; Xiaobo et al., 2010). The influence of muscle pigments and water content on the classification of chicken breast fillets was also highlighted by Yang et al. (2018), where samples were successfully classified in different quality grades.

The b coefficients of PLS-R models (Fig. 10) for FT-IR spectral data revealed the important contribution of certain wavelengths in model development. Absorption bands at 1011, 1032 and 1111-1143 ​cm^-1^ were related to polyglycines, polysaccharides (C–O stretch) and amines (NH_2_ rock/twist), respectively (Bocker et al., 2007). Specifically, the absorption at 1032 ​cm^-1^ which corresponds to polysaccharides, could be associated to biofilm formation by *Pseudomonas* spp. on stored chilled meat (Liu et al., 2015; Wickramasinghe et al., 2019, 2020). Additionally, high absorption occurred in the regions of 1222–1230 ​cm^-1^, 1284-1289 ​cm^-1^ and 1345-1352 ​cm^-1^ which are linked to the presence of lipids, nucleic acids (asym PO_2_-stretch), amines from free amino acids and amide III (Argyri et al., 2014). The critical role of amides and free amines for the prediction of spoilage in meat is presented via high b coefficients at 1369- 1426 ​cm^-1^ and 1464- 1567 ​cm^-1^ (Böcker et al., 2007). These outcomes are in compliance to the existing literature where absorption bands of 1650, 1550 and 1400- 1200 ​cm^-1^ are linked to amide I, II and III and subsequently to the proteolytic activity of *Pseudomonas* spp. on meat (Nychas and Tassou, 1997; Ellis et al., 2002). Especially for chicken breast analysis via FTIR and NIR spectroscopy, the estimation of spoilage in intact chicken breast muscle was influenced by the absorption bands at 1080, 1550 and 1640 ​cm^-1^ and the increased content in free amino acids and peptides as a result of proteolysis during storage maintenance (Alexandrakis et al., 2012). In another study the estimation of microbial spoilage was attempted at 600- 1110 ​cm^-1^ where the findings indicated the region of 1000–1060 ​cm^-1^ corresponding to protein functional group, such as R-CO-NH_2_, R-NH_2_,R-CO-NH-R and R-NH-R as the most significant (Lin et al., 2004).

## 5 Conclusion

The results of multivariate data analysis showed the impact of variation among and within batches on model performance and subsequently the important role of data selection for model development and validation. Moreover, the perplexity of choosing the suitable machine learning approach and the necessity of comparison between models was underlined in order to select the most accurate approach. FT-IR analysis was proved to be the most appropriate technique for the assessment of spoilage on the surface of chicken breast fillets while lars and nnet algorithms predicted satisfactorily the microbial loads of TVC on the surface of chicken breast fillets. Further on, additional experimental data as well as validation with data from dynamic storage conditions simulating the distribution stage could lead to the improvement of the abovementioned models.

## Funding

This research has been co-financed by the European Regional Development Fund of the European Union and Greek national funds through the Operational Program Competitiveness, Entrepreneurship and Innovation, under the call RESEARCH-CREATE-INNOVATE (project code: T1EDK-04344, “A Model Smart Quality Assurance and Safety System for Fresh Poultry Products-QAPP”).

## CRediT authorship contribution statement

**Evgenia D. Spyrelli:** Methodology, Formal analysis, Data curation, Validation, Writing – original draft. **Onur Ozcan:** Methodology, Software, Formal analysis, Data curation, Visualization. **Fady Mohareb:** Software, Formal analysis, Data curation, Supervision, Visualization. **Efstathios Z. Panagou:** Investigation, Supervision, Validation, Writing – review & editing. **George- John E. Nychas:** Conceptualization, Funding acquisition, Resources, Project administration, Reviewing and Editing.

## Declaration of competing interest

The authors declare that they have no known competing financial interests or personal relationships that could have appeared to influence the work reported in this paper.
