# Supplementary material for: Spoilage assessment of chicken breast fillets by means of fourier transform infrared spectroscopy and multispectral image analysis
Source: Curr Res Food Sci. 2021 Feb 25;4:121–31. doi: 10.1016/j.crfs.2021.02.007 (PMC7961306; doi:10.1016/j.crfs.2021.02.007)

**Supplementary material**

**Tables:**

Table A: Kinetic parameters of the primary growth model of Baranyi and Roberts (1994) for TVC and *Pseudomonas* spp.

|  |  | **lag (h)** | **μ_max_ (h^-1^)** | **y_o_**  **(log CFU/cm^2^)** | **y_max_**  **(log CFU/cm^2^)** | **se(fit)** | **R^2^** |
| --- | --- | --- | --- | --- | --- | --- | --- |
|  | TVC | 92.6 | 0.0382 | 2.7 | 5.5 | 0.433- 0.492 | 0.826- 0.879 |
| **0 ^o^C** | *Pseudomonas* spp. | 72.2 | 0.0356 | 2.1 | 5.2 | 0.497- 0.514 | 0.826- 0.877 |
|  | TVC | 52.8 | 0.0570 | 3.0 | 6.6 | 0.429- 0.551 | 0.875- 0.937 |
| **5 ^o^C** | *Pseudomonas* spp. | 17.5 | 0.0610 | 2.0 | 6.1 | 0.304- 0.522 | 0.866- 0.944 |
|  | TVC | 22.7 | 0.0903 | 3.1 | 6.8 | 0.279- 0.506 | 0.894- 0.964 |
| **10 ^o^C** | *Pseudomonas* spp. | Ν/Α | 0.0991 | 2.3 | 6.4 | 0.242- 0.489 | 0.899- 0.979 |
|  | TVC | 10.2 | 0.2141 | 2.9 | 6.8 | 0.224- 0.545 | 0.879- 0.982 |
| **15 ^o^C** | *Pseudomonas* spp. | 8.8 | 0.2410 | 1.9 | 6.7 | 0.282- 0.442 | 0.941- 0.978 |

Ν/Α: not available; y_o_: initial microbial load in sample (log CFU/cm^2^); y_max_: maximum microbial load in sample (log CFU/cm^2^); se(fit): standard error of fit;

**Figure caption:**

Fig. A: Composition (%) of gases (O_2_: blue line, CO_2_: red line) in packaged chicken breast fillet samples during storage at 0, 5, 10, and 15 ^o^C.


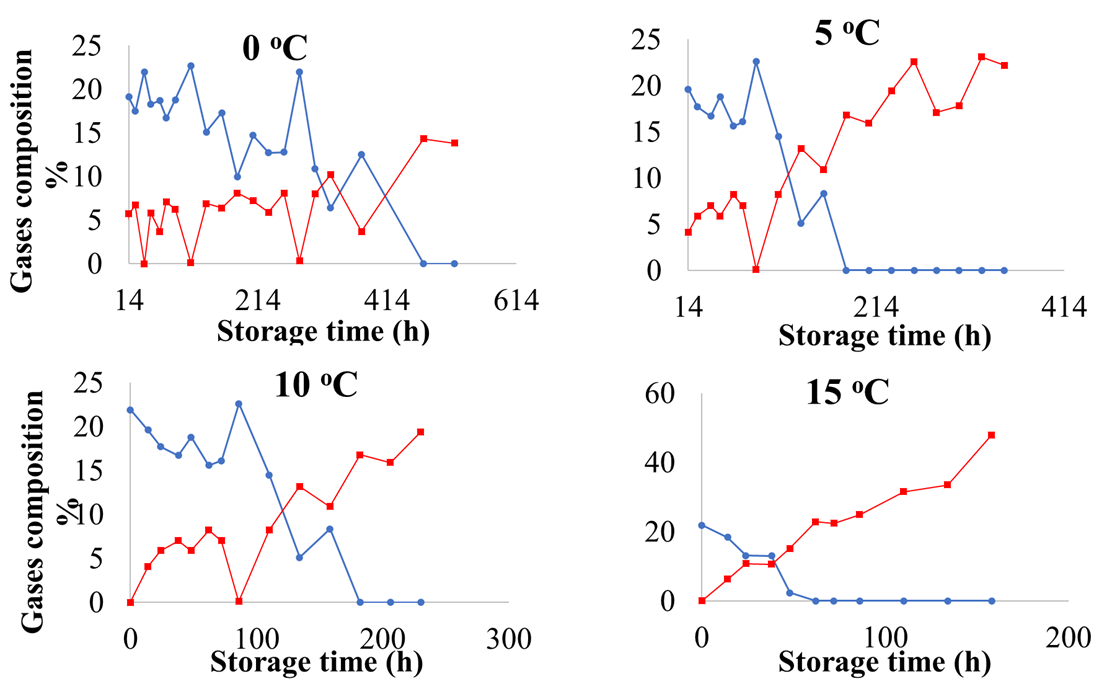

Supplement: Multimedia component 1 [file mmc1.docx]
